# Regulation of Hippo/YAP signaling and Esophageal Squamous Carcinoma progression by an E3 ubiquitin ligase PARK2

**DOI:** 10.7150/thno.46078

**Published:** 2020-07-25

**Authors:** Xiaofeng Zhou, Yajie Li, Weilong Wang, Sujie Wang, Jinghan Hou, Aijia Zhang, Benjie Lv, Can Gao, Ziyi Yan, Dan Pang, Kui Lu, Nor Hazwani Ahmad, Lidong Wang, Jian Zhu, Lichen Zhang, Ting Zhuang, Xiumin Li

**Affiliations:** 1 Xinxiang Key Laboratory for Molecular Therapy of Cancer, Xinxiang Medical University, Xinxiang 453003, Henan Province, P.R. China.; 2 Department of Gastroenterology, the Third Affiliated Hospital of Xinxiang Medical University, Xinxiang 453003, Henan Province, P.R. China.; 3 Oncological and Radiological Sciences, Advanced Medical and Dental Institute, Universiti Sains Malaysia, Bertam, 13200 Kepala Batas, Penang, Malaysia.; 4 Henan Key Laboratory of immunology and targeted therapy, School of Laboratory Medicine, Henan Collaborative Innovation Center of Molecular Diagnosis and Laboratory Medicine, Xinxiang Medical University, Xinxiang, 453003, Henan Province, P.R. China.; 5 Xinxiang Key Laboratory of Tumor Migration, Invasion and Precision Medicine, Xinxiang, 453003, Henan Province, P.R. China.; 6 School of Laboratory Medicine, Xinxiang Medical University, Xinxiang, 453003, Henan Province, P.R. China.; 7 State Key Laboratory of Esophageal Cancer Prevention & Treatment and Henan Key Laboratory for Esophageal Cancer Research of The First Affiliated Hospital, Zhengzhou University, Zhengzhou 450052, China.; 8 Department of General Surgery, The Second Hospital, Cheeloo College of Medicine, Shandong University, Shandong Province, P.R. China.

**Keywords:** PARK2, Hippo, YAP, ESCC, Ubiquitin

## Abstract

**Objective:** Esophageal squamous cell carcinoma (ESCC) is one of the most commonly diagnosed cancer types in China. Recent genomic sequencing analysis indicated the over-activation of Hippo/YAP signaling might play important roles for the carcinogenic process and progression for ESCC patients. However, little is known about the molecular mechanisms that controls Hippo signaling activity in ESCC. Our previous studies indicated that PLCE1-an important risk factor for ESCC-linked to ESCC progression through snail signaling, during this period, we found PARK2 was an important downstream target of PLCE1-snail axis. PARK2 was decreased in ESCC human samples, and correlated with good prognosis in ESCC patients. Further research showed that PARK2 could inhibit YAP, which functions as key downstream effectors of the Hippo pathway. Here, we aim to reveal the molecular mechanisms of PARK2 modulated Hippo pathway in ESCC.

**Methods:** To evaluate the function of PARK2 in ESCC, we used a tissue microarray (TMA) of 223 human ESCC patients and immunohistochemistry to analyze the correlation between PARK2 expression and clinicopathologic variables. Depletion of endogenous PARK2 and YAP from ESCC cells using CRISPR/Cas9 technologies. Flow cytometry and EdU cell proliferation assay were used to detect proliferation of ESCC cells. Nude mice subcutaneous injection and Ki-67 staining were used to evaluate tumor growth *in vivo*. Migration and invasion assays were performed. In addition, lung metastasis models in mice were used to validate the function of PARK2 *in vivo*. Identification of PARK2 involved in hippo pathway was achieved by expression microarray screening, double immunofluorescence staining and co-immunoprecipitation assays. The RNA-seq analysis results were validated through quantitative real-time PCR (qRT-PCR) analysis. The protein half-life of YAP was analyzed by Cycloheximide assay, and the TEAD activity was detected by Luciferase reporter assays.

**Results:** Clinical sample of ESCC revealed that low PARK2 expression correlated with late tumor stage (P < 0.001), poor differentiation (P < 0.04), lymph node (P < 0.001) and distant metastasis (P = 0.0087). Multivariate Cox proportional regression analysis further revealed that PARK2 expression (P = 0.032) is an independent prognostic factor for the overall survival of ESCC patients. Besides, the immunohistochemistry results showed that PARK2 negatively correlated with YAP protein level (P *<* 0.001). PARK2 depletion promotes ESCC progression both through Hippo/YAP axis, while PARK2 overexpression suppresses ESCC tumor progression by Hippo signaling. Co-IP and ubiquitination assays revealed that PARK2 could interact with YAP in the cytosol and promotes YAP K48-linked ubiquitination at K90 sites.

**Conclusion:** Clinical sample analysis and mechanistic study have validated PARK2 as a tumor suppressor for ESCC. Multivariate Cox proportional regression analysis further revealed that PARK2 is an independent prognostic factor for the overall survival of ESCC patients. Cellular and molecular mechanisms in this study showed that PARK2 associated with YAP protein in the cytosol, promoted YAP ubiquitination and proteasome-dependent degradation in ESCC cells. Therefore, as a novel modulator for Hippo signaling, modulation of PARK2 activity or gene expression level could be an appealing strategy to treat esophageal.

## Introduction

Esophageal cancer is the eighth most common malignancy of cancer incidence and mortality worldwide 1. Among all the esophageal cancer patients, about 60% of them are diagnosed in China. According to cancer statistics, over 300,000 new esophageal cancer cases happen in China 2. Esophageal cancer in China exhibits a different pathological pattern compared with western countries, while esophageal squamous cell carcinoma (ESCC) is the major subtype. Besides, even with a high incidence of ESCC in China, there are dramatic high district variations in China mainland. Some districts, such as the northern part of Henan province, have higher ESCC incidence 3. Besides the environmental and living habit related factors, including tobacco and alcohol consumption, recent studies through genomic sequencing showed that genetic alternations, such as gene mutations and amplifications, are pervasive in human ESCC samples and play important roles in carcinogenesis 4. Interestingly, the genomic sequencing data showed that the deficiency of inhibitory factors of Hippo/YAP pathway, such as AJUBA and FATs mutations, or YAP gene amplification accounted for 48% of ESCC samples, indicating that dys-regulation of Hippo signaling could play critical role in ESCC progression 5. However, the insights of the molecular mechanisms that control Hippo signaling activity and YAP/TEAD turnover are of utmost importance for ESCC diagnostics and therapeutics.

The Hippo signaling was initially identified from Drosophila 6. Further studies showed that Hippo signaling is an evolutionarily conserved pathway, which modulates tissue growth and organ size in a range of species. The core of Hippo signaling is composed of a kinase cascade: the upstream phosphorylation kinase MST1/2 promotes LATS1/2 phosphorylation and activation, which subsequently phosphorylates the pathway effector YAP/TAZ and promotes YAP/TAZ retention in the cytosol and degradation 7. But, If YAP/TAZ are not phosphorylated, they will trans-locate into the nuclear, interact with transcriptional factors, such as TEAD and RUNX to regulate genes involved in cell growth, migration, survival and metabolism^8^-^10^. Not surprisingly, dys-regulation of Hippo signaling has been implicated in many human cancers, including esophageal cancer 11, 12. Several components of Hippo signaling are found mutated in ESCC, including FATs, AJUBA, STK3, LATS1 and DCHS1 5, 13. Besides, YAP gene amplification and elevated expression are also observed in ESCC 14. Based on these findings, we can observe several possible or confirmed mechanisms that lead to the inappropriate YAP/TEAD activation in ESCC, such as mutations of the inhibitory factors of Hippo signaling or elevated YAP expression 15. However, as obvious candidates emerge components of ubiquitin-proteasome system, having been shown to safeguard Hippo signaling and modulating cancer progression.

PARK2 (Parkin) was firstly found as the Parkinson disease-related gene 16. In such a neurodegenerative disease, the mutations of PARK2 gene were common in Juvenile Parkinson disease 17. Further studies revealed that park2 deficiency causes mitochondrial disorder and oxidative stress in neurons 18, 19. Besides, PARK2 forms an interesting regulation loop with P53 signaling, in which P53 promotes PARK2 gene expression, while PARK2 in turn inhibits P53 signaling through promoting P53 protein degradation 20, 21. PARK2 is found to diminished expression in several human cancers 22-25. However, high up to 90% of P53 gene was mutated in ESCC, indicating PARK2 not likely effects through P53 signaling in ESCC. Thus, the mechanism that PARK2 contributes to tumor suppression and the regulation of PARK2 in ESCC are not clear in ESCC.

Here, we demonstrated PARK2 as an important inhibitor for Hippo/YAP signaling. PARK2 was decreased in ESCC human samples, correlated with good prognosis in ESCC patients and negatively related to YAP expression. PARK2 inhibited ESCC cancer progression through Hippo/YAP axis. PARK2 was found to associate with YAP in the cytosol and promoted YAP K48-linked ubiquitination and degradation. Hence, PARK2 functions to safeguard hippo signaling activity in ESCC, which could be a promising marker for ESCC cancer diagnostics and therapeutics.

## Results

### PARK2 functions as a tumor suppressor in ESCC

**Figure 1:**
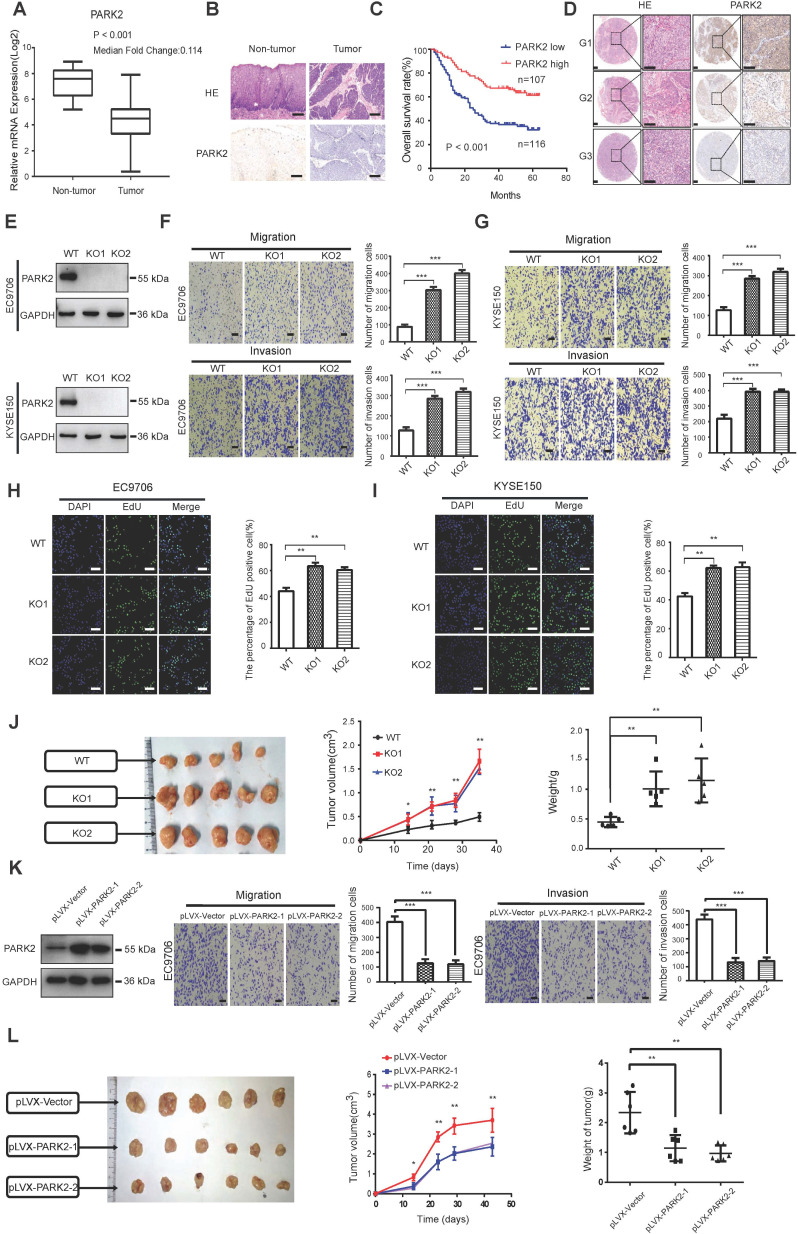
** The implications of PARK2 in human ESCC samples and its effect on cancer-related phenotype in ESCC cell lines. A.** PARK2 mRNA levels were significantly decreased in ESCC compared with matched adjacent non-tumor tissues. The data were obtained from TCGA database. **B.** PARK2 protein expression was significantly decreased in ESCC tissues compared with their adjacent non-tumor tissues as analyzed by IHC. **C.** Kaplan-Meier analysis revealed that low PARK2 expression was related with poorer overall survival of ESCC patients. P < 0.001, log-rank test. **D.** H & E staining was used to show different differentiation statuses of the ESCC (G1: high differentiation;G2: middle differentiation;G3:low differentiation). **E.** Immuno-blots showing CRISPR-mediated deletion of PARK2 in ESCC cell lines. **F and G.** PARK2 knockout promoted the migration and invasion in EC9706 cells (F) and KYSE150 cells (G) as determined by transwell assays. **H and I.** EC9706 cells (H) and KYSE150 cells (I) were labeled with EdU. EdU-positive cells, green; cell nuclei, blue; scale bar 100 µm. **J.** PARK2 knockout promoted the tumor growth of EC9706 cells in a xenograft model. The growth of xenografts was monitored over 5 weeks. Xenograft tumors were then dissected and their weights determined. **K.** Representatives and summary of migration and invasion assay showing that overexpression of PARK2 inhibited cell migration and invasion in EC9706 cells. **L.** PARK2 overexpression delayed the tumor growth of EC9706 cells in a xenograft model.

We firstly analyzed the expression of esophageal cancer from TCGA database (https://tcga-data.nci.nih.gov/tcga/). The public available data indicated that PARK2 decreased eight folds in the esophageal cancer compared with normal esophageal tissue (Figure 1A). In order to confirm this finding, we investigated ESCC samples together with adjacent esophageal tissues and observed that PARK2 expression is significantly decreased in ESCC tumors (Figure 1B). Based on 223 ESCC patients sample analysis by IHC (immunohistochemistry), we found that low PARK2 expression correlated with late tumor stage (*P* < 0.001), poor differentiation (*P* < 0.04) (Figure 1D), lymph node (*P* < 0.001) and distant metastasis (*P* = 0.0087) (Table 1). Besides, cox analysis showed that several risk factors related to poor prognosis in ESCC patients, including poor differentiation (*P* = 0.015), tumor invasion depth (*P* = 0.001), lymph node metastasis (*P* < 0.001), distant metastasis (*P* < 0.001) and low PARK2 expression (*P* < 0.001) (Table 2). KMPLOT survival analysis showed that low PARK2 expression significantly correlated with poor prognosis (*P* < 0.001) (Figure 1C).

We further investigated the function of PARK2 in two ESCC cell lines (EC9706 and KYSE150). We generated stable clones of PARK2 knocking-out cell lines originated from EC9706 and KYSE150 cells (Figure 1E). We measured the migration and invasion capacity by trans-well assays with permeable filter and basement membrane respectively. The trans-well assays demonstrated that PARK2 KO cells increased the migration and invasion capacity in both EC9706 and KYSE150 cell models (Figure 1F-G). The wound healing assays showed similar results that PARK2 KO cells showed increased wound closure speed compared with wild type cells in both EC9706 and KYSE150 cell models (Figure S4A-B). The cell growth assay showed that PARK2 KO cells dramatically increased the number of EdU positive cells compared with wild type cells in both EC9706 and KYSE150 cells (Figure 1H-I). The *in vivo* tumor growth assay showed that PARK2 KO cells increased growth speed in EC9706 cell models (Figure 1J). Besides, we generated the PARK2 overexpression cell lines by lenti-virus infection. PARK2 overexpression inhibited cell migration and invasion in EC9706 cells (Figure 1K). The wound-healing assay showed that PARK2 overexpression inhibited the wound-healing speed (Figure S4C). The clone formation assay showed that PARK2 overexpression inhibited the clone formation capacity in EC9706 cells (Figure S4D). The *in vivo* tumor growth assay showed that PARK2 overexpression significantly inhibited tumor growth in xenograft mice models (Figure 1L).

### PARK2 inhibits ESCC progression through Hippo/YAP axis

**Figure 2:**
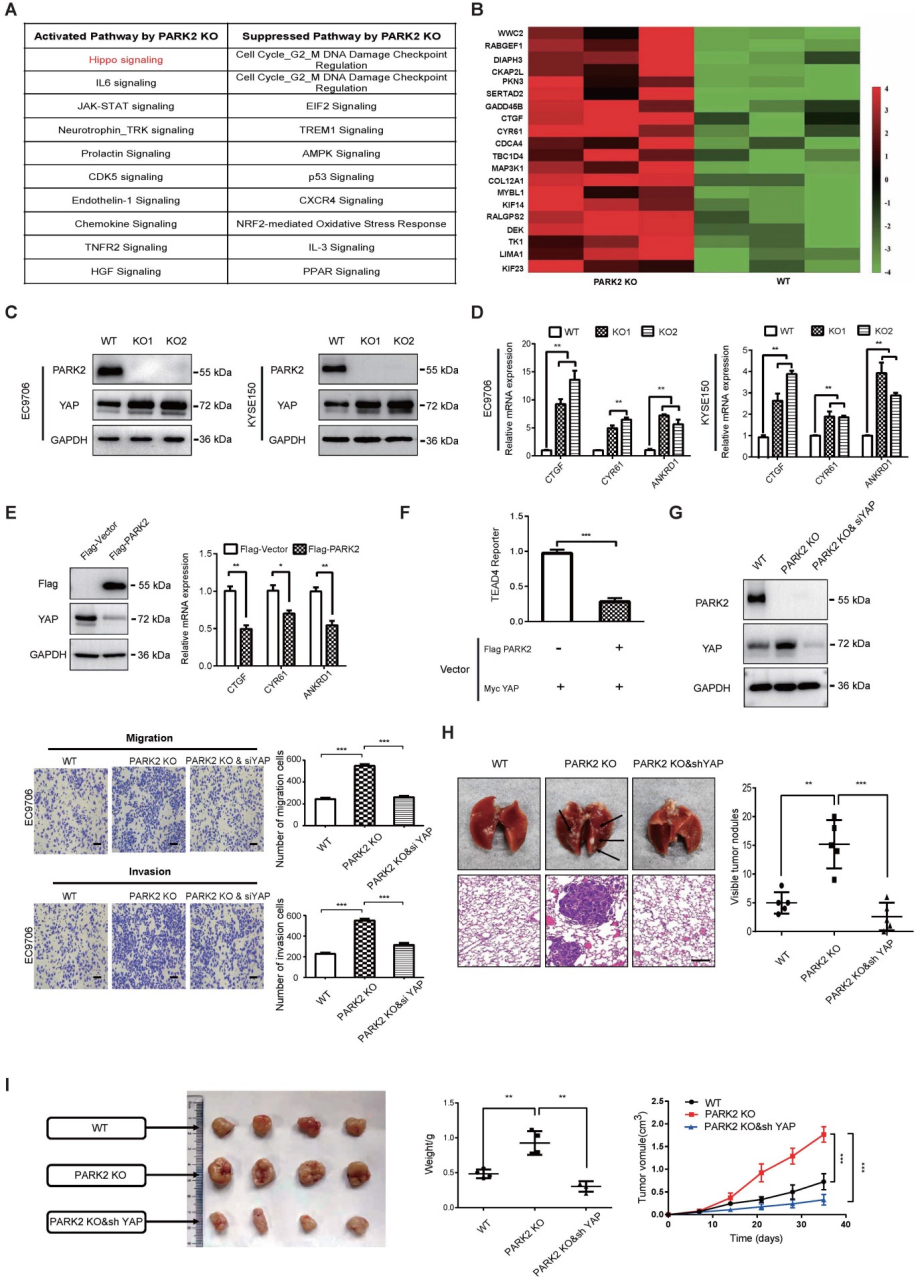
** PARK2 inhibits ESCC progression by Hippo signaling. A.** KEGG pathway assay of differential mRNA transcripts in PARK2 KO clones identified by RNA-seq. **B.** Heat map of mRNA changes in WT and PARK2 KO single clones of EC9706 by bulk RNA-seq. **C.** Western blotting assays of PARK2 in WT and PARK2 KO single clones of EC9706 and KYSE150 cells. **D.** PARK2 KO increased mRNA expression of Hippo target genes in cells. **E.** Flag-PARK2 expression reduced mRNA expression of Hippo target genes and YAP protein level in EC9706 cells. **F.** Flag-PARK2 expression inhibited TEAD luciferase reporter activities in EC9706 cells. **G.** Knockdown of YAP rescued the migration and invasion ability of cells with the PARK2 knockout. Scale bar; 100 µm. **H.** Knockdown of YAP rescued metastasis ability of cells with the PARK2 knockout *in vivo*. Black arrow indicates the pulmonary metastatic nodule. Scale bar, 100 µm. **I.** Knockdown of YAP rescued xenograft tumor growth of cells with the PARK2 knockout *in vivo*. **P < 0.01, ***P < 0.001.

In order to investigate the potential mechanism, we carried out RNA sequencing analysis in PARK2 WT and KO cells. The signaling pathway analysis showed that PARK2 KO activated several pathways, such as Hippo signaling and IL-6 pathway, while inhibited several tumor suppressor pathways, including AMPK signaling and P53 signaling (Figure 2A). Figure 2B showed that a group of Hippo signaling target genes were increased in PARK2 KO cells, such as CTGF and CYR61 (Figure 2B). The immuno-blotting analysis showed that PARK2 KO increased YAP protein level in EC9706 and KYSE150 cells (Figure 2C), while the classical Hippo/YAP target genes were increased by PARK2 knocking-out in both EC9706 and KYSE150 cells, such as CTGF and CYR 61 (Figure 2D). We further analyzed the other Hippo pathway components, including TAZ, LATS1/2 and MST1/2. The immuno-blotting data showed that PARK2 knocking-out did not change these protein levels (Figure S5D). Consistently, PARK2 overexpression inhibited YAP protein level and Hippo target gene expression (Figure 2E). The luciferase assay showed that PARK2 overexpression inhibited TEAD responsive element activity (Figure 2F).

In order to investigate the logic link between the cancer phenotype and Hippo/YAP signaling in PARK2 function, we carried out several rescue experiments. In figure 2G, PARK2 knocking-out in EC9706 cells could promote cell migration and invasion, which effect could be at least partial rescued by further YAP knocking down. However, YAP knocking-out could inhibit cell migration and invasion, which effect could not be further rescued by PARK2 knocking-out (Figure S5C and Figure S3). These data indicated that PARK2 modulated Hippo signaling through YAP protein. The *in vivo* metastatic assay showed that PARK2 KO could promote lung metastasis in mice, which effect could be rescued by YAP knocking down (Figure 2H). The xenograft tumor assay showed that YAP knocking down could rescue the increased tumor growth speed by PARK2 knocking out (Figure 2I).

### PARK2 expression reversely relates with YAP level in human samples and interacts with YAP protein in the cytosol

**Figure 3:**
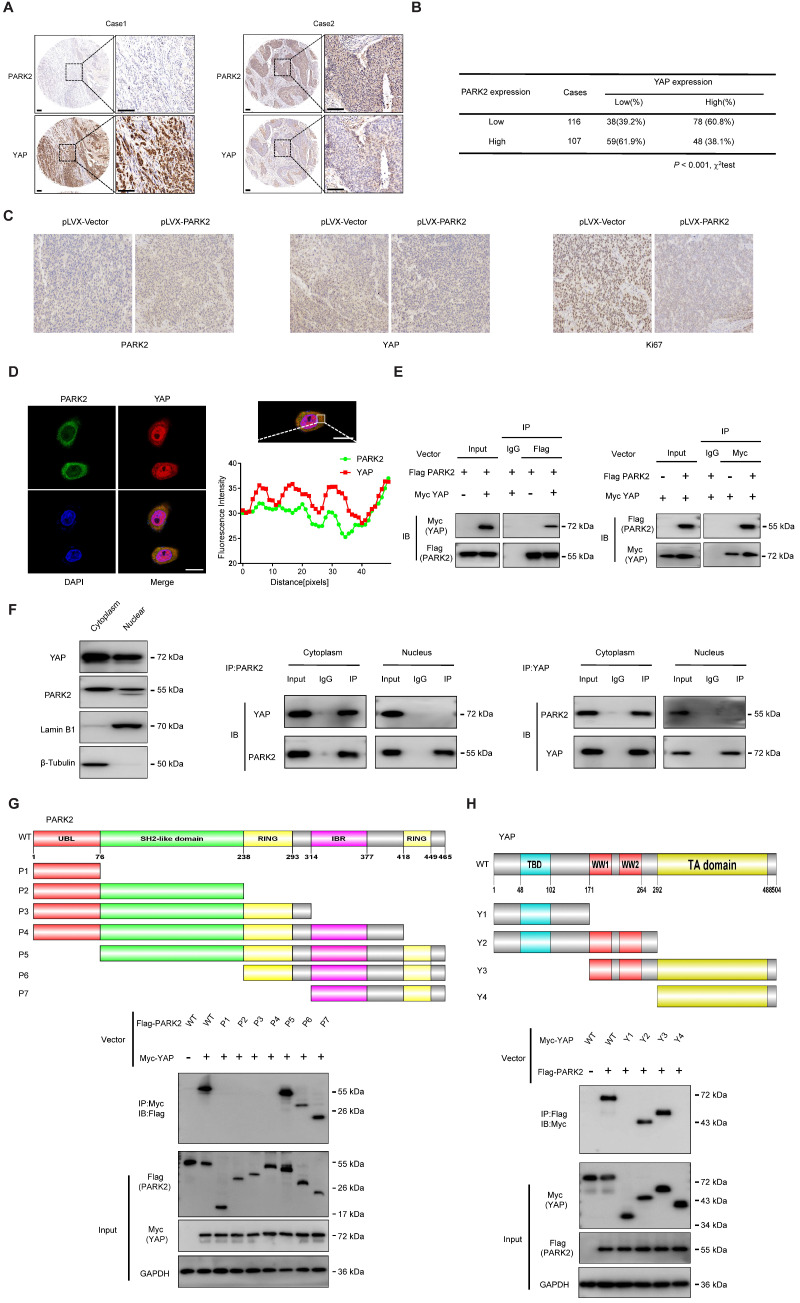
** PARK2 expression is negatively correlated with YAP and interacts with YAP in ESCC cells. A and B.** Low PARK2 expression was significantly correlated with increased YAP levels in ESCC specimens analyzed by IHC staining. Scale bar, 100 µm. **C.** The negative correlation between YAP and PARK2 expression in xenograft tumors, which is analyzed by IHC staining. Scale bar, 100 µm. **D.** IF showed that co-localization of PARK2 (red) and YAP (green). Nuclei were stained with DAPI (blue). Scale bar, 20 µm. The fluorescence-integrated density was measured by ImageJ software; while the mander's co-localization coefficients were measured by Zen software. The mean co-localization coefficient=0.77± 0.03. **E.** PARK2 interacted with YAP in ESCC cells. **F.** PARK2 is mainly localized in the cytoplasm. The subcellular protein fractionation kit was used for cytoplasm and nuclear separation. Tubulin and Lamin B1 were used for cytoplasm and nuclear control. PARK2 interacted with YAP in cytoplasm. **G.** PARK2 bound to YAP at its Ring domain. (Top panel) Schematic representation of vectors expressing Flag-tagged wild-type or serial deletion mutants of PARK2. (Bottom panel) The Ring domain of PARK2 interacted with YAP. **H.** YAP bound to PARK2 at its WW domain. (Top panel) Schematic representation of vectors expressing Myc-tagged wild-type or serial deletion mutants of YAP. (Bottom panel) The WW domain of YAP interacted with PARK2.

We further analyzed the expression level of PARK2 and YAP in human ESCC samples. The immunohistochemistry results showed that PARK2 negatively correlated with YAP protein level (P < 0.001) (Figure 3A-B). Besides, such trend in YAP and PARK2 expression was also observed in xenograft tumor samples (Figure 3C). Immuno-staining showed that PARK2 was localized mainly in the cytosol, while YAP was located both in the cytosol and nuclear (mean co-localization coefficient=0.77± 0.03) (Figure 3D). Immuno-precipitation assay showed that YAP could interact with PARK2 (Figure 3E). Further cell fraction separation assay showed that YAP interacted with PARK2 in the cytosol (Figure 3F). In order to investigate the interaction domain between the two proteins, we made the sub-clone variants of PARK2 and YAP (Figure 3G-H). The immuno-precipitation assay showed that the RING domain at the C-terminal of PARK2 was required for its interaction with YAP, while the WW domain of YAP was responsible to associate with PARK2 (Figure 3G-H).

### PARK2 modulates YAP stability by ubiquitination dependent manner

**Figure 4:**
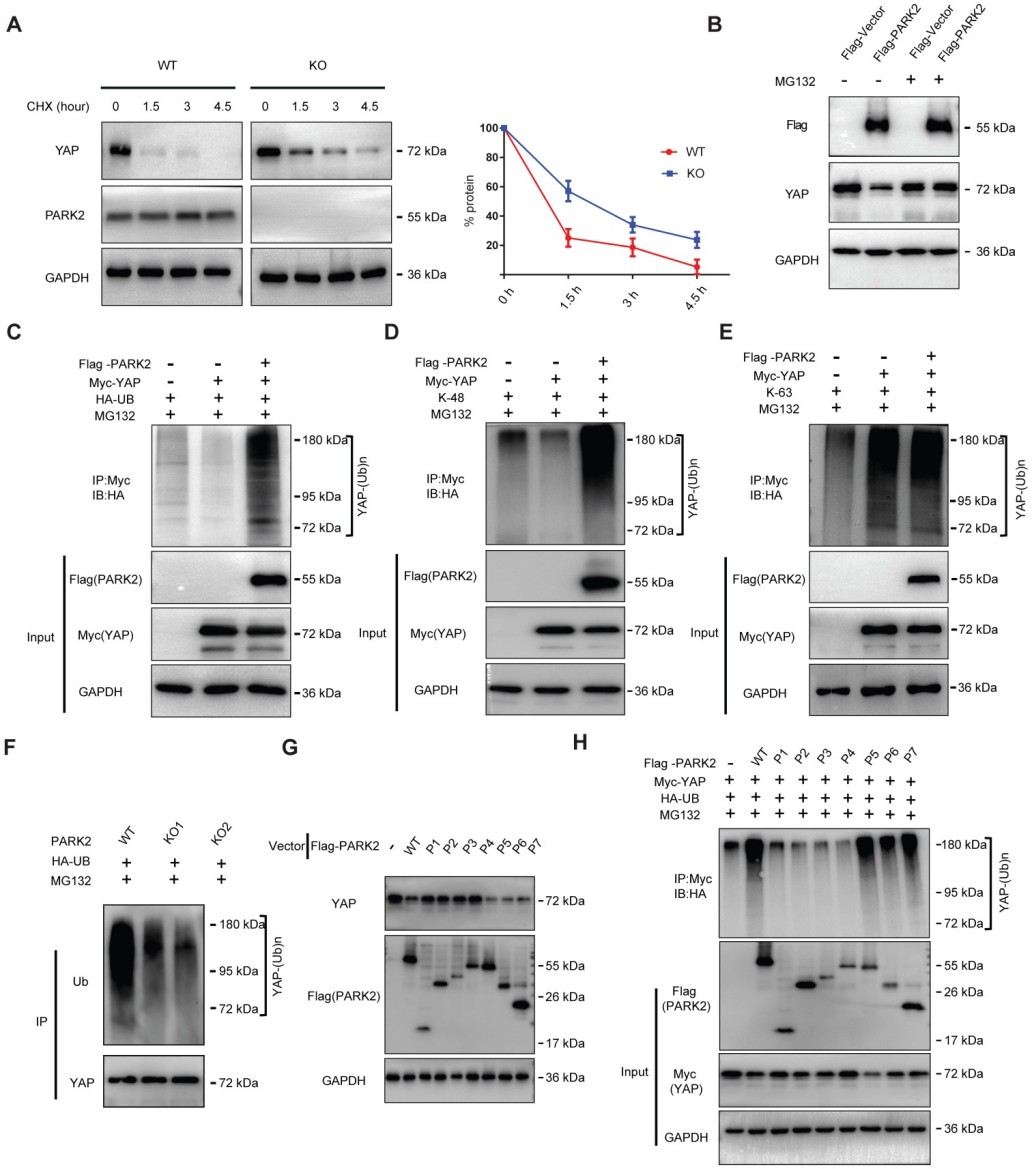
** PARK2 promotes YAP protein degradation through ubiquitination. A.** PARK2 knockout decreased YAP protein half-life in EC9706 cells. The cells were treated with 100 μmol/L CHX for indicated time periods before being collected for western-blot assays. **B.** PARK2 over-expression could inhibit YAP protein level, which effect could be diminished by MG132. **C.** Ubiquitin-based Immuno-precipitation showed that PARK2 promoted YAP overall poly-ubiquitination in HEK293T cells. **D.** Ubiquitin-based Immuno-precipitation showed that PARK2 promoted YAP K48-linked ubiquitinaiton in HEK293T cells. **E.** Ubiquitin-based Immuno-precipitation showed that PARK2 did not affect YAP K63-linked ubiquitinaiton in HEK293T cells. **F.** Ubiquitin-based immuno-precipitation showed that PARK2 KO inhibited endogenous YAP overall poly-ubiquitination in EC9706 cells. **G.** The RING domain of PARK2 is required for PARK2 to YAP protein suppression. **H.** The RING domain of PARK2 is required for PARK2 to regulate ubiquitination of YAP.

There are two possible regulatory mechanisms for PARK2 to regulate YAP expression level, which could be transcriptional regulation or post-translational regulation. The QPCR data indicated no statistical difference of YAP mRNA level between PARK2 WT and KO groups (Figure S5A). Since PARK2 functions as an E3 ubiquitin ligase, it is most likely for PARK2 to modulate YAP through ubiquitin-based manner. We firstly investigated the PARK2 effect on YAP stability by cycloheximide, which indicated that PARK2 KO significantly prolonged the half-life of YAP in EC9706 cells (Figure 4A). However, the proteasome inhibitor MG132 could diminish the inhibition effect of YAP protein by PARK2 over-expression, which indicated such regulation was proteasome dependent (Figure 4B). We further investigated the effect of PARK2 on YAP protein ubiquitination. The ubiquitination-based IP showed that PARK2 could promote the overall ubiquitination level and K48-linked ubiquitination level of YAP, but did not change the K63-linked ubiquitination of YAP (Figure 4C-E). We further investigated the overall ubiquitination level of YAP in EC9706 cells. The ubiquitination-based IP showed that PARK2 knocking-out significantly decreased endogenous YAP poly-ubiquitination (Figure 4F).

This conclusion was confirmed by the ubiquitination-based IP assay in denatured conditions (Figure S5F). However, our ubiquitination assay showed that purified PARK2 could not directly ubiquitinate YAP alone (Figure S5B). We further investigated the functional domain of PARK2 to induce YAP poly-ubiquitination. Figure G and H indicated that the RING domain of PARK2 is necessary for YAP protein poly-ubiquitination and degradation (Figure 4G-H).

### PARK2 facilitates YAP protein ubiquitination at K90 site, which effect depends on PARK2 E3 ligase activity

**Figure 5:**
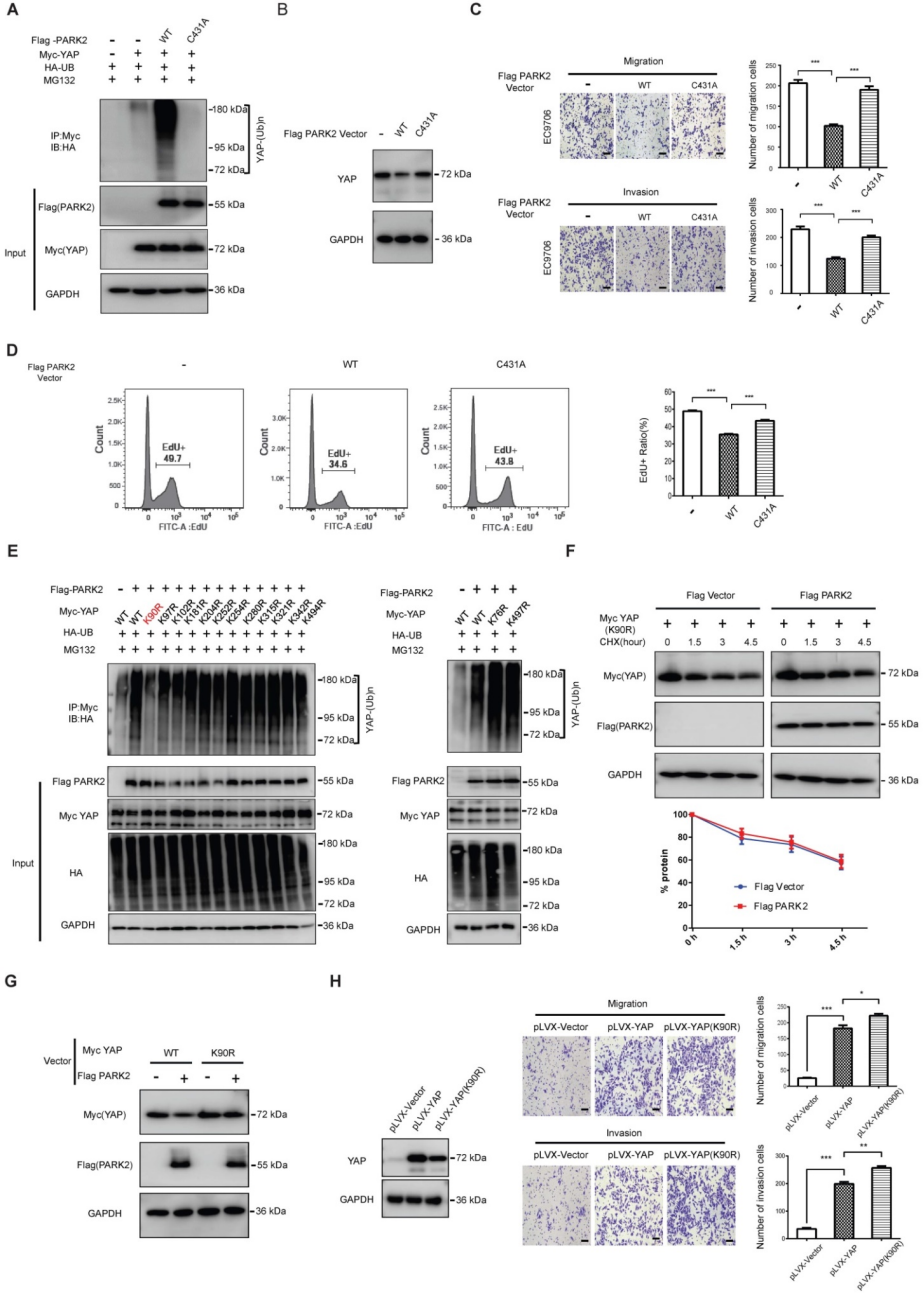
** PARK2 promotes YAP poly-ubiquitination at K90 site and depends on its ubiquitin ligase activity. A.** The effects of expression of Flag PARK2 and its mutants on ubiquitination of Myc YAP in 293T cells analyzed by *in vivo* ubiquitination assays. **B.** Mutations of PARK2 that impaired PARK2's ubiquitination activity impaired the ability of PARK2 to degrade YAP protein in EC9706 cells. **C.** Mutations of PARK2 that impaired PARK2's ubiquitination activity rescued the migration and invasion ability of EC9706 cells with the overexpression of wild type PARK2. Scale bar, 100 µm. **D.** Mutations of PARK2 that impaired PARK2's ubiquitination activity rescued the proliferation ability of EC9706 cells with the overexpression of wild type PARK2. **E.** K90 mutation (K90R) largely abolished ubiquitination of YAP by PARK2. 293T cells were transfected with indicated vectors for *in vivo* ubiquitination assays. **F.** PARK2 could not further decrease YAP (K90R) half-life in HEK293 cells. The cells were treated with 100 μmol/L CHX for indicated time periods before being collected for western-blot assays. **G.** PARK2 could inhibit YAP WT protein level, but had no effect on YAP (K90R) mutant variant. **H.** YAP (K90R) overexpression could have stronger phenotype in cancer cell migration and invasion in EC9706 cells.

We further mutated the C431 sites of PARK2, which is required for PARK2 E3 ligase activity. The ubiquitination-based assay showed the PARK2^C431A^ mutant form could not inhibit YAP protein level or induce the poly-ubiquitination of YAP in ESCC cells (Figure 5A-B). Besides, the migration and invasion trans-well assay showed that PARK2 WT could inhibit the migration and invasion of EC9706 cells, while the PARK2^C431A^ mutant form could not (Figure 5C). The EdU-based flow-cytometry showed that PARK2 wild type form could inhibit ESCC cell proliferation, while the PARK2^C431A^ mutant form showed little effect on cell proliferation (Figure 5D). We further investigated the exact ubiquitin ligation sites of YAP by PARK2. Since YAP protein has 13 lysine sites, we made 13 mutant variants of YAP. The ubiquitin-based IP showed that PARK2 promoted YAP polyubiquitination mainly at K90 site (Figure 5E). Further experiments showed that YAP^K90R^ form was resistant to PARK2 degradation effects (Figure 5F-G). Interestingly, the YAP^K90R^ form showed longer half-life compared with YAP^WT^ in HEK293 cells (Figure S5E). Besides, lenti-virus based infection with YAP^WT^ and YAP^K90R^ in EC9706 cells showed that cells infected with YAP^K90R^ conferred stronger invasive phenotype in EC9706 cells compared with YAP^WT^ cells (Figure 5H).

## Discussion

**Figure 6:**
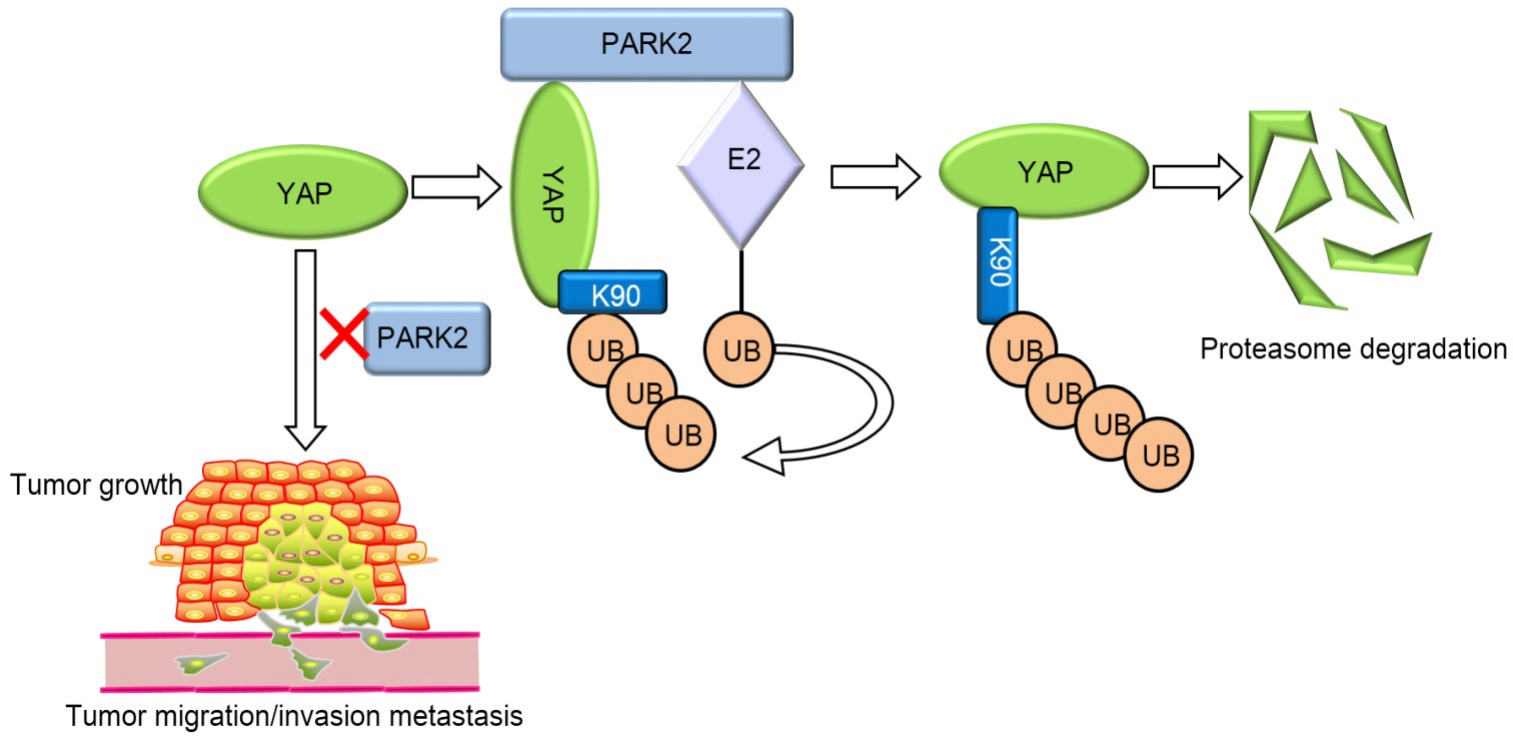
PARK2 associates with YAP, promotes YAP K48-linked ubiquitination and degradation in ESCC cells, which inhibits the activation of Hippo/YAP axis and ESCC cancer progression.

Our current study reports a famous RING family E3 ligase PARK2 functions as an endogenous inhibitor for HIPPO/YAP axis in ESCC cancer. PARK2 is dramatically decreased in human ESCC samples, relates to good prognosis in ESCC patients and correlates with YAP protein negativity by IHC staining. PARK2 inhibits ESCC cancer cell progression both in cell culture and mice models. Mechanism study shows that PARK2 promotes YAP K48-linked poly-ubiquitination and proteasome-dependent degradation in ESCC cells (Figure 6). Based on these data, we can propose that the modulation of PARK2 expression or PARK2 activity could a strategy to treat YAP-driven ESCC patients.

The conserved Hippo pathway controls tissue hemostasis and organ size in several species 26. Since Hippo signaling is an inhibitory pathway, the dys-function of Hippo inhibitors, such as AJUBA and FATs mutation, or Hippo signaling effector YAP/TAZ overexpression, which causes Hippo/YAP axis over-activation, could play critical roles in carcinogenic process of several human cancers 5. Interestingly, one genomic-based study showed that the mutation rate of Hippo signaling ranks NO. 8 among all cancer-related pathways, which might indicate the genomic events of Hippo signaling abnormality are common in human cancer. In addition, one genomic sequencing study based on Chinese ESCC patient samples revealed that ESCC genomic abnormality could be clustered into five pathways, including cell cycle signaling, histone modification pathway, Hippo signaling, Notch signaling and PI3K pathway 20. The genomic abnormity events of Hippo signaling were existed in almost half of ESCC patients, which implicated the important role of Hippo signaling in ESCC carcinogenic process and cancer progression. Several molecular mechanism studies showed that YAP depletion inhibited ESCC cell migration and invasion 27, 28. Since ESCC is such dependent on Hippo signaling, the Hippo pathway could an “Oncogenic addiction” pathway for ESCC. YAP/TAZ could be promising drug target for ESCC cancer therapeutics, while understanding the potential mechanism how YAP protein is regulated might provide novel insight into ESCC treatment.

It has been well established that Hippo signaling activity is mainly regulated through controlling the function of Hippo effectors-YAP/TAZ 11. The function of YAP is mainly regulated through its trans-location between the cytosol and the nuclear. The phosphorylation by the Hippo pathway kinases cascade retains YAP protein in the cytosol and promotes YAP protein degradation, whereas the upstream pathways could compromise the YAP phosphorylation level, which permits YAP to enter the nuclear and activate Hippo target genes 6, 12. However, the recent studies showed that the ubiquitin-proteasome system is important safeguard to prevent over-activation of human cancers. For example, SCF^b-TRCP^ complex is important for YAP protein polyubiquitination and degradation 29, while our recent study showed that SHARPIN-the LUBAC (linear ubiquitination assembly complex) component, could also facilitate YAP protein K48-linked poly-ubiquitination and degradation in ESCC cells 30. Our current study revealed a novel layer of Hippo pathway regulator-PARK2, which associated with YAP at its C-terminal, promoted YAP poly-ubiquitination and degradation, which controlled the YAP protein half-life, turnover and also the duration of Hippo signaling output in ESCC. In consistent, PARK2 decreased its expression eight folds compared with normal esophageal tissue. Besides, PARK2 related to good prognosis, Well-differentiation of ESCC and YAP protein negativity in ESCC samples. Coupled with these data, we can assume that loss of PARK2, which subsequently lead to Hippo/YAP over-activation, could be a critical factor for ESCC carcinogenesis and progression.

PARK2 (also named Parkin) is composed of four functional domains, including UBL domain, RING0 (unique PARKIN domain), RING1, IBR domain (RING-in-between-RING domain) and RING2 domain 31. PARK2 has been shown to function as an E3 ubiquitin ligase and controls several stress response pathway, such as mitochondrial quality control and autophagy 32, 33. One interesting finding in PARK2 field is that the mutation of PARK2 genes are related with hereditary early onset Parkinson disease (PD) 34. However PARK2 was observed to decrease in several human cancers, including glioblastoma, ovary cancer, lung cancer and breast cancer 22, 23, 35, 36. It is also intriguing that patients with Parkinson disease have higher risk of other cancers, such as melanoma, which might indicate the involvement of PARK2 deletion/mutation in the carcinogenesis process 37. Several mice model studies showed that PARK2 KO mice are susceptible to hepatocellular carcinoma and colon cancer, suggesting that PARK2 could be important contributor for oncogenic process 22, 38. However, our previous studies indicated that PLCE1-a important risk factor for ESCC-linked to ESCC progression through snail signaling 39, 40. Interestingly, our unbiased RNA sequencing data between PLCE WT and PLCE knockout cells revealed that PARK2 was important downstream target of PLCE1-snail axis 39. Not surprisingly, further investigation showed PARK2 was a tumor suppressor in ESCC from clinical aspects, while the molecular mechanisms revealed a novel regulatory manner: PARK2 modulated Hippo pathway by promoting YAP protein ubiquitination and degradation in ESCC cells. Hence, PARK2 expression or mutation status could be an interesting prediction or prognosis marker for ESCC cancer patients.

In conclusion, we have validated PARK2 as a tumor suppressor for ESCC both in clinical sample and experimental studies. We demonstrated that PARK2 depletion was pervasive in ESCC and related to poor survival. PARK2 associated with YAP protein in the cytosol, promoted YAP ubiquitination and proteasome-dependent degradation in ESCC cells. Our studies revealed a novel function of PARK2 in Hippo signaling in multiple layers. As a novel modulator for Hippo signaling, modulation of PARK2 activity or gene expression level could be an appealing strategy to treat esophageal cancer.

## Materials and Methods

### Cell culture

Esophageal carcinoma cell lines (EC9706 and KYSE150) were cultured in RPMI 1640 Medium (Biological Industries) containing 10% fetal bovine serum (Biological Industries), 1% penicillin/streptomycin (Invitrogen). HEK-293 cells were cultured in DMEM Medium supplemented with 10% FBS and 1%penicillin/streptomycin. EC9706 and KYSE150 cells were authenticated by short tandem repeat profiling (STR). STR profiling of our KYSE150 cells was found to be 100% consistent with the STR data of the KYSE150 from China Infrastructure of Cell Line Resources (Figure S1). EC9706 cell STR profiling data was not accessible in public databases including ATCC. Cells were regularly tested for mycoplasma using Lookout Mycoplasma PCR detection kit (MP0035, Sigma) and only used when negative.

### Generation of knockout

The PARK2 sgRNA was designed, synthesized, and cloned to the pX460 cloning vectors. Then, 100 million ESCC cells were electroporated with 4 μg of pX460 plasmid containing sgRNA using the Nucleofector™ 2b Device (Lonza). After the electroporation, cell population was sorted by flow cytometry. Single cell can be obtained and then added into 96-well plates. Further 10 days' culture was allowed for the single cell clone expansion. The clones were collected for PCR amplification and sequencing to analyze the gene mutation in the PARK2 sgRNA or YAP sgRNA recognizing site. The PARK2 sgRNA sequences or siRNA sequence were shown in Table S1. The PARK2 KO sequence was shown in Figure S2. The YAP KO sequence was shown in Figure S3.

### RNA isolation and quantitative real-time PCR (qRT-PCR)

Total RNA was extracted with RNeasy Plus Mini Kit (Qiagen, Valencia, CA) following the manufacturer's specifications. Reverse transcription was performed using the RevertAid First Strand cDNA Synthesis Kit (Thermo, Lithuania). qRT-PCR was carried out using GoTaq® qPCR Master Mix (Promega, USA) and 7500 Fast Real-Time PCR System (Applied Biosystems, Singapore). GAPDH was used as an internal control. The sequence of the primers for qPCR was listed in Table S2.

### Western blot

Standard western-blot assays were used to analyze protein expression in cells. The following antibodies were used for assays: anti-Flag-M2 (A8592, Sigma, 1:1000), anti-HA (2013819001, Roche, 1:1000) anti-Myc (9E10, Santa Cruz, 1:1000), anti-GAPDH (0411, Santa Cruz, 1:1000), anti-PARK2 (Abcam, ab15954), anti-YAP (63.7, Santa Cruz, 1:1000), Protein signals were detected with an ECL kit ( Millipore Co., Billerica, Massachusetts, USA).

### Immunofluorescence (IF) staining

Cells on the coverslips were fixed with 4% paraformaldehyde and incubated with the primary antibody against PARK2 (Santa cruz, sc-32282), YAP (CST, 14074) at 4 °C overnight. After washing with PBS, cells were then incubated with fluorescence-conjugated secondary antibody (Invitrogen, Carlsbad, CA), and subsequently counterstained with DAPI (Life Technology). Images were captured after staining with anti-fade DAPI solution using a confocal laser-scanning microscope (Leica TCS SP8 STED). The fluorescence-integrated density was measured by ImageJ software and the mander's co-localization coefficients were generated by Zen software.

### Co-IP assays

Co-IP assays were performed according to standard protocols. For the co-IP of Flag-PARK2 and Myc-YAP proteins, anti-Flag (A2220, Sigma) and anti-Myc (9E10, Santa Cruz) agarose beads (30 µl) were used to pull down Flag-PARK2 and Myc-YAP, respectively. Beads were washed with PBST three times, and bound protein was denatured with 2× SDS sample buffer. The supernatants were collected and proceeded to SDS-PAGE western blot analysis.

### Wound healing and Transwell assays

For the wound healing assay, cells were cultured in 6-well plates until confluent and then wounded with a sterile tip. The cells were captured at the indicated time points after scratching. The distances between the two edges of the scratched wound were measured using Image J software. The trans-well system (8 μm pore size, Corning) was employed for cell migration and invasion assays. For migration assays, cells in serum-free medium were seeded into the upper chambers. For invasion assays, the upper chambers were coated with matrigel (BD Biocoat, USA).After 24 h, cells that had migrated through to the bottom of the insert membrane were fixed, stained with crystal violet and counted under ×20 objective lens. The experiments were repeated thrice.

### *In vivo* ubiquitination assays

For *in vivo* ubiquitination assays, cells were transfected with vectors, including expressing Myc-YAP, Flag-PARK2 and HA-Ub, respectively, for 24 h. Cells were then treated with MG132 (10 μM) for 6 h, and the levels of Myc-YAP ubiquitination was determined by IP with an anti-Myc antibody followed by western-blot assays with an anti-HA antibody (2013819001, Roche, 1:1000).

### Ubiquitination assay in purified proteins

Ubiquitination was analyzed with an ubiquitination kit (Boston Biochem) following protocols recommended by the manufacturer. Recombinant proteins were mixing with 20X E1 Enzyme, 10X Mg2+-ATP Solution, 10X Ubiquitin Solution, 1ug E2 Enzyme (UbcH7, Boston Biochem; UBE2D1, Sino Biological Inc.) in a final volume of 20 µl reaction buffer. The reaction was carried out at 37 °C for 1 h and products were analyzed by western-blot assays with anti-YAP antibody (#14074, Cell signaling, 1:1000).

### *In vivo* tumorigenesis and metastasis assay

For *in vivo* tumorigenic experiment, EC9706 cells (4×10^6^) were injected into the right dorsal flank of 4-week-old female BALB/c nude mice. Tumor formation in nude mice was monitored over a 4-week period. The tumor volume was calculated by the formula: tumor volume = 0.5 × length × width^2^ .For *in vivo* metastasis assays, each experimental group consisted of 5 4-week-old female BALB/c nude mice. Briefly, 2×10^6^ cells were injected intravenously through the tail vein into mouse. The mice were killed 8 weeks after injection. Tumor nodules formed on the lung surfaces were macroscopically determined and counted. The lungs were excised and embedded in paraffin. Further, the tissue sections were stained with H&E to visualize the structure.

### Luciferase reporter assays

For TEAD luciferase activity assays, cells with ectopic Flag-PARK2 expression and their control cells were transfected with the TEAD luciferase reporter vector for 24 h. Cells were then harvested for assays. Luciferase reporter assays were performed using the dual luciferase assay kit (Promega). The pRL-null vector expressing renilla luciferase (Promega) was used as an internal control to normalize the transfection efficiency.

### Cycloheximide assay

Cycloheximide was added into culture medium with the final concentration of 100 μmol/L. Cell lysis were collected at 1.5, 3 and 4.5h after the treatment of cycloheximide.

### Tissue microarray (TMA) and immunohistochemistry (IHC)

223 cases of ESCC were selected for the TMA construction. All of these tissue samples were obtained from the First Affiliated Hospital of Xinxiang Medical University. All patients signed informed consent. No patients recruited in the study received preoperative treatments. The ESCC samples used in this study were authorized by the Committees for Ethical Review of Research at Xinxiang medical University. IHC was performed according to a standard streptavidin-biotin-peroxidase complex method Signals in tumor cells were visually quantified using a scoring system from 0 to 9. The scores were obtained by multiplying the intensity of signals with the percentage of positive cells (signal: 0 = no signal, 1 = weak signal, 2 = intermediate signal, and 3 = strong signal; percentage: 0 = 0%, 1 ≤ 25%, 2 = 25-50%, and 3 ≥ 50%). Low and high expression were defined as scores of < 6 and ≥ 6, respectively.

### Cell proliferation assay

Cell proliferation was assessed by EdU incorporation and flow cytometry. Proliferating cells were determined by using the 5-ethynyl-20-deoxyuridine (EdU) assay kit (Ribobio, Guangzhou, China). For quantification analysis of the images, each data point represents the positive fluorescence area calculated from a minimum of five randomly chosen fields from three individual experiments. EdU incorporation flow cytometry assay was carried out according to the manufacturer's instructions. The experiments were performed in triplicate.

### Statistical analysis

No specific statistical tests were used to predetermine the sample size. Statistical analysis was performed using GraphPad Prism 7 software or SPSS version 23.0 (SPSS, Inc., IL). Data were expressed as mean ± S.E.M (standard error of the mean). Differences between two independent groups were tested with Student's t-test. Kaplan-Meier analysis with log-rank test was applied for survival analysis. The relation between PARK2 expression and clinicopathological characteristics was analyzed by Pearson χ^2^ test. Univariate and multivariate Cox proportional hazard regression models were used to evaluate the survival hazard using Cox proportional hazard model with a forward stepwise procedure. Differences were considered to be statistically significant when *P* < 0.05 (*P < 0.01; **P < 0.001).

## Supplementary Material

Supplementary figures and tables.

## Figures and Tables

**Table 1:**
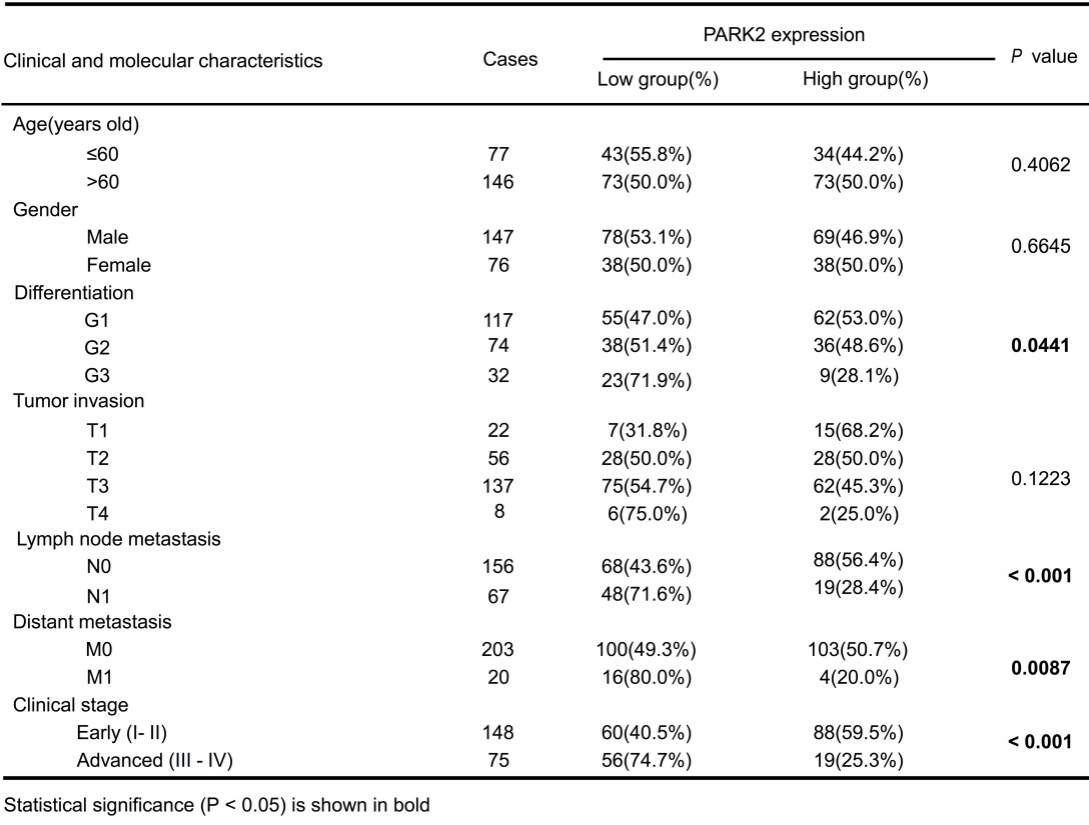
Clinicopathological correlation of PARK2 expression in ESCC

**Table 2:**
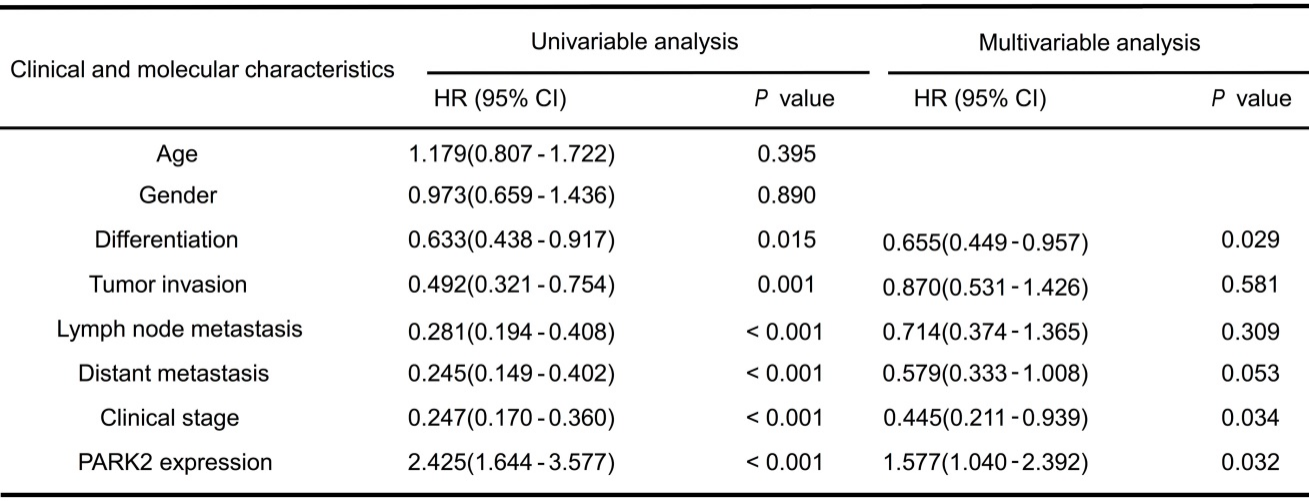
Cox proportional hazard regression analyses for overall survival

**Table 3:**
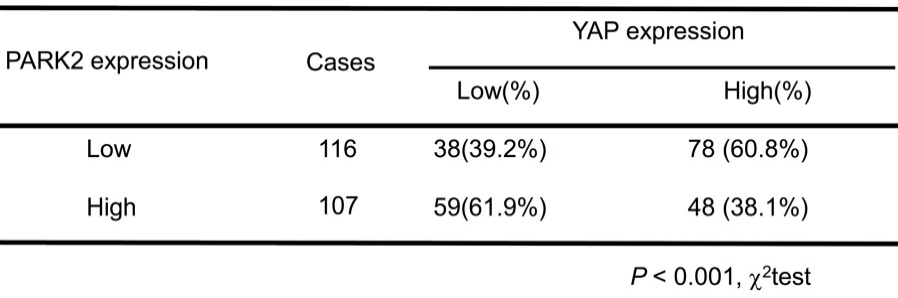
Association of PARK2 expression and YAP expression in ESCC

## Abbreviations

ESCC: esophageal squamous cell carcinoma
YAP: yes-associated protein
TEAD: TEA domain transcriptional factor
RING: really interesting new gene
LATS: large tumor suppressor kinase
PARK2: Parkinson disease protein 2
