# Supplementary material for: Regulation of Hippo/YAP signaling and Esophageal Squamous Carcinoma progression by an E3 ubiquitin ligase PARK2
Source: Theranostics. 2020 Jul 25;10(21):9443–57. doi: 10.7150/thno.46078 (PMC7449928; doi:10.7150/thno.46078)
Supplement: Supplementary file 1 — Supplementary figures and tables. [file thnov10p9443s1.pdf]

**Supplementary Figure legends:**

**Figure S1:** The STR profiling of our KYSE150 cells
**Figure S2:** The sequencing of PARK2 KO in EC9706 and KYSE150 cells.
**Figure S3:** The sequencing of YAP KO in EC9706 cells.
**Figure S4: A and B:** PARK2 knockout increased the wound-healing ability in EC9706 cells and KYSE150 cells compared with the wild type cells. **C:** Overexpression of PARK2 inhibited the wound-healing ability compared with control cells in EC9706 cells.**D:** Colony formation ability decreased in PARK2 overexpression cells compared with control cells in EC9706 cells.
**Figure S5: A:** PARK2 knockout did not increase YAP mRNA level in EC9706 cells. **B:** *In vitro* ubiquitination assay showed that PARK2 could not induce the poly-ubiquitination of YAP, while the FBXW7 was used as the positive control for YAP ubiquitination. **C:** YAP KO could reduce cell migration and invasion capacity in EC9706 cells, which effect could not be rescued by further PARK2 knockout. **D:** PARK2 knockout did not change the protein levels of other Hippo signaling components, including TAZ, LATS1/2, MST1/2 in EC9706 and KYSE150 cells. **E:** YAP K90R mutant was more stable compared with YAP WT form. **F:** PARK2 could facilitate YAP poly-ubiquitination in HEK293 cells.

**Table S1:** The primer sequences for Real-time PCR
**Table S2:** Small interfering RNA sequence for YAP and PINK1
**Table S3:** The guide RNA sequence for PARK2 and YAP knockout

# Supplementary Figure 1

KYSE150

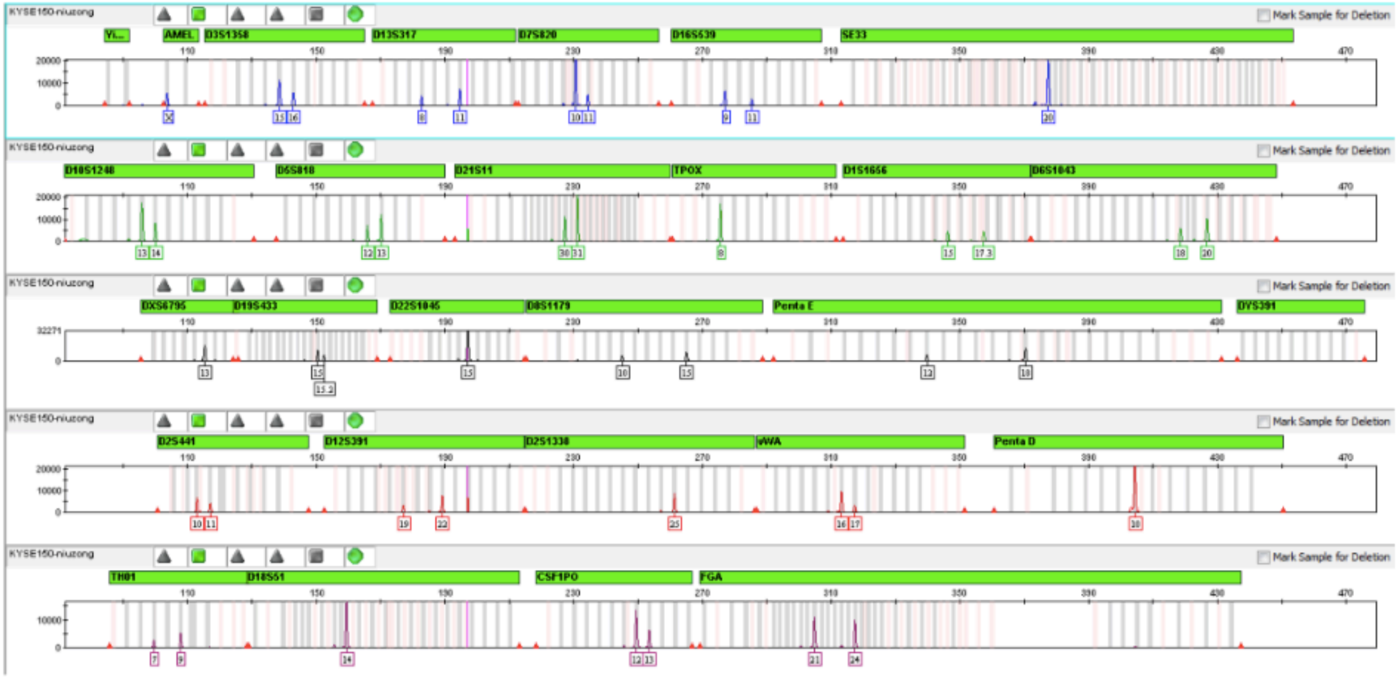


| EV | Cell No. | Cell name | Locus names | | | | | | | | |
|---|---|---|---|---|---|---|---|---|---|---|---|
| | | | D5S818 | D13S317 | D7S820 | D16S539 | VWA | TH01 | AM | TPOX | CSF1PO |
| | Query (Your Cell) | | 12,13 | 8,11 | 10,11 | 9,11 | 16,17 | 7,9 | X,X | 8,8 | 12,13 |
| 1.00(36/36) | 375 | KYSE-150 | 12,13 | 8,11 | 10,11 | 9,11 | 16,17 | 7,9 | X,X | 8,8 | 12,13 |

# Supplementary Figure 2

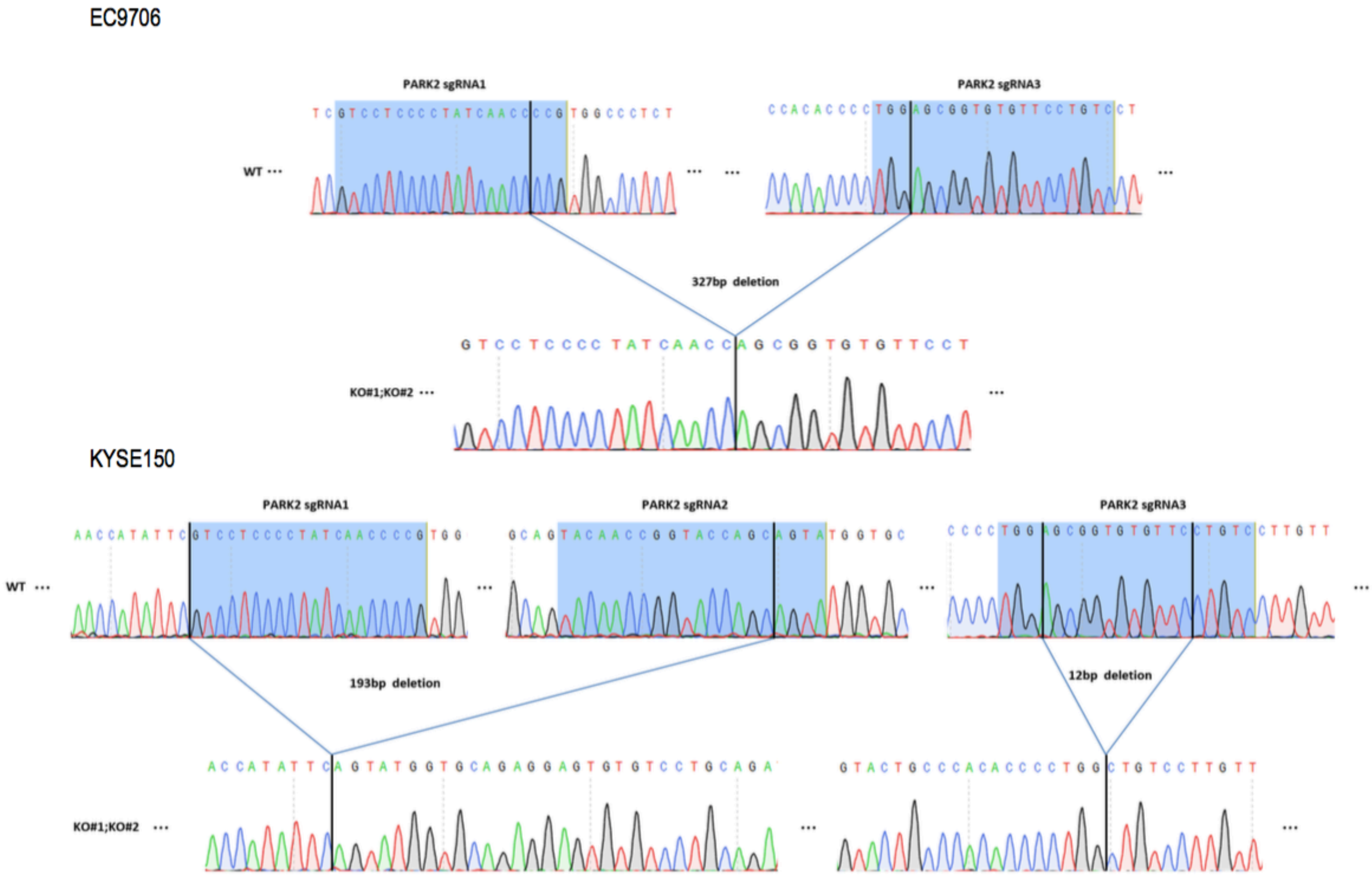

# Supplementary Figure 3

## EC9706

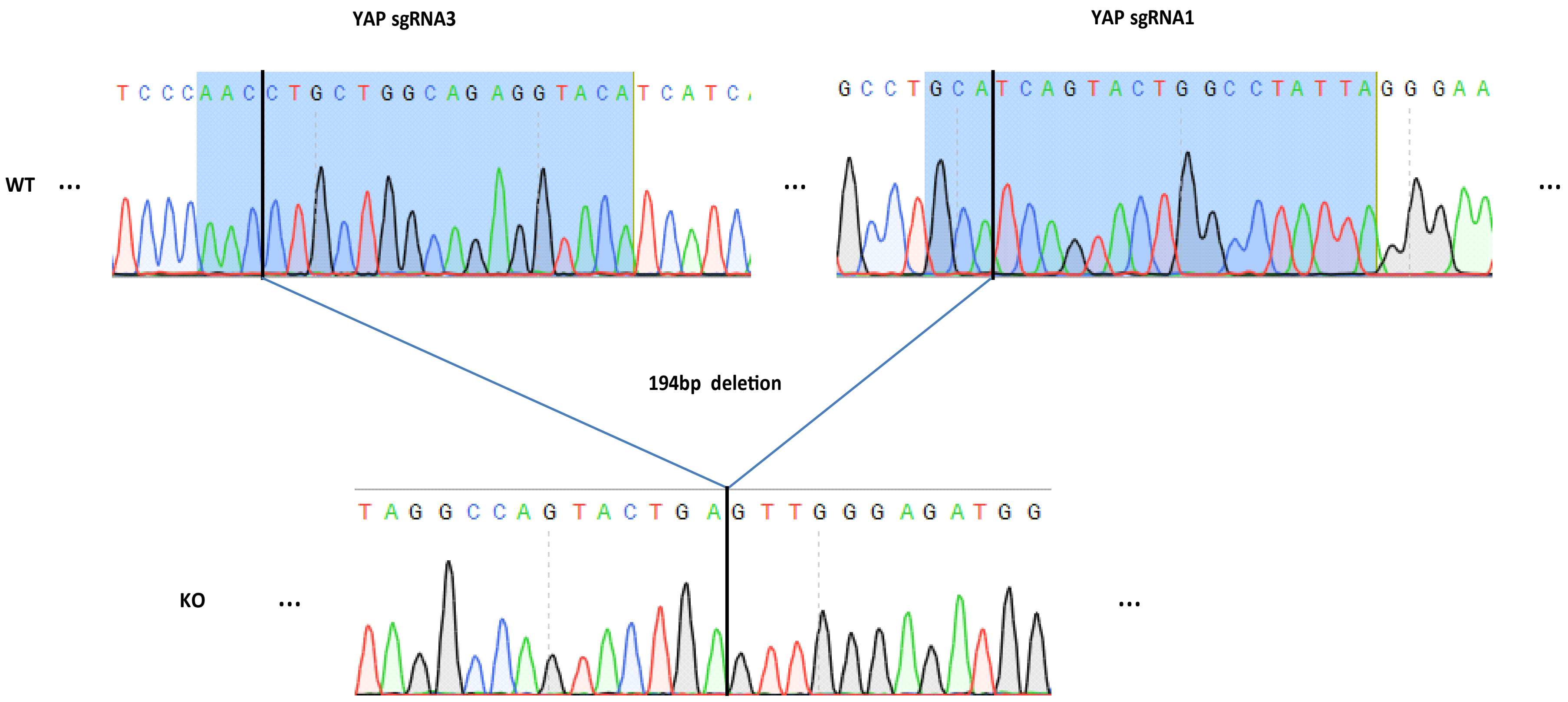

Supplementary Figure 4

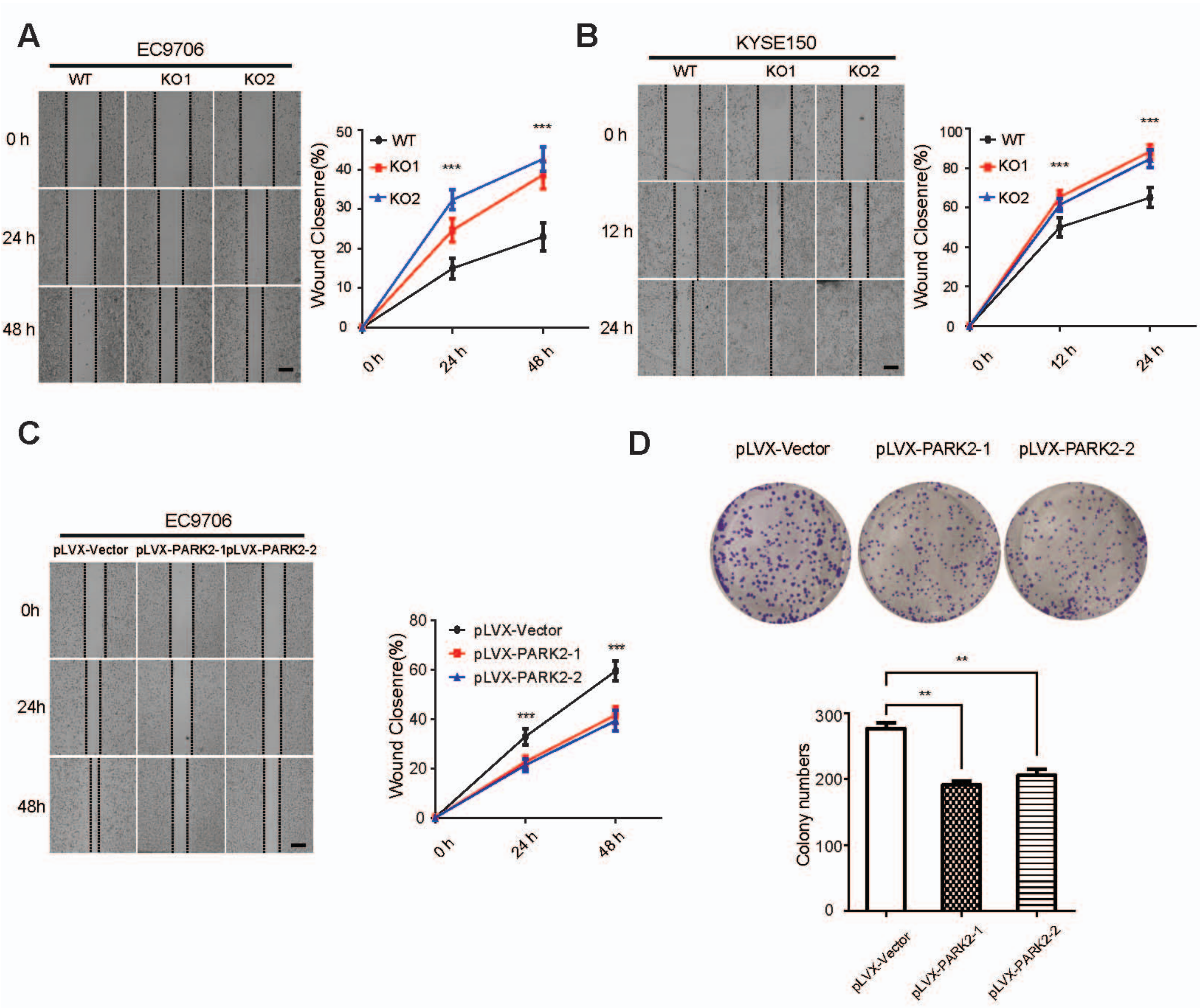

# Supplementary Figure 5

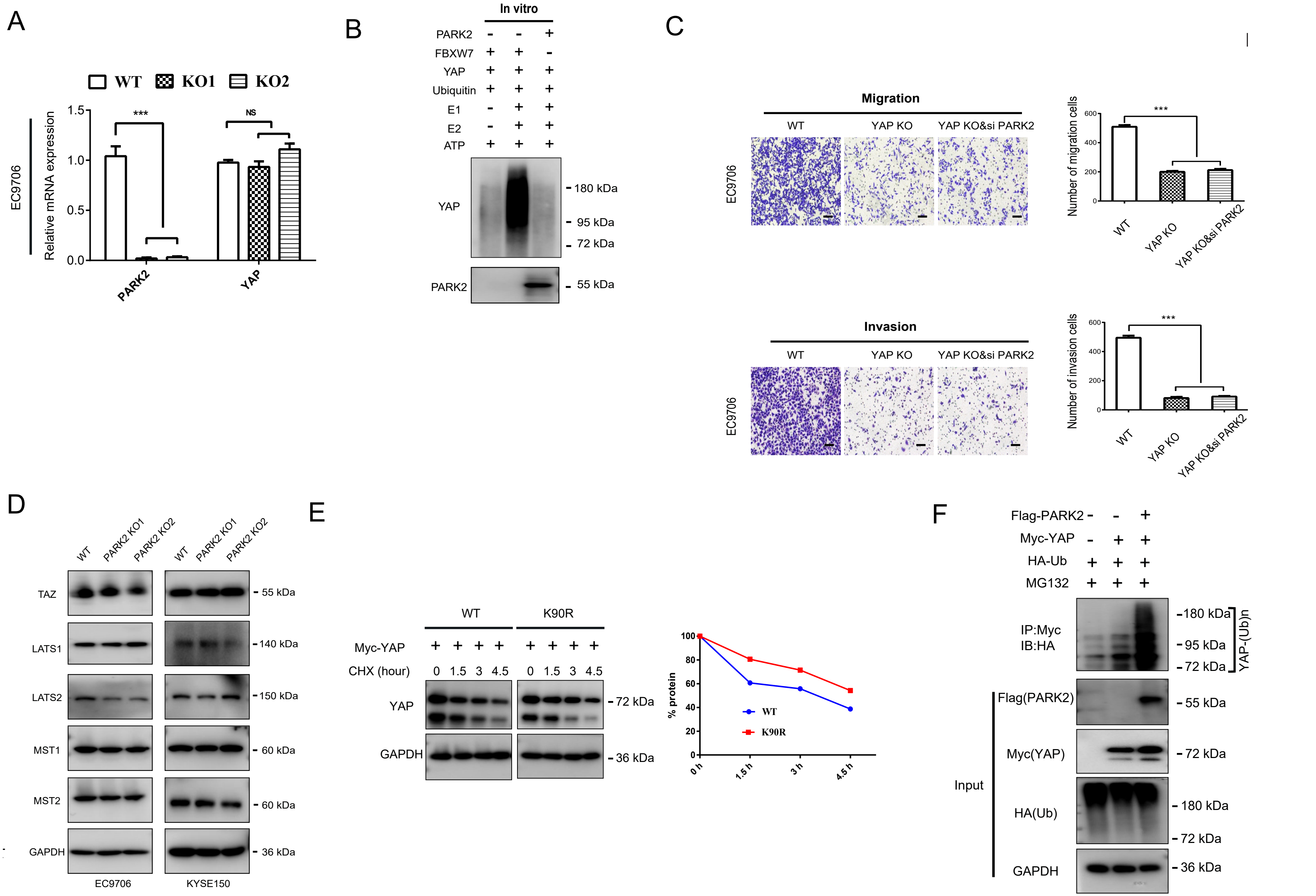

Supplementary table 1

Primer sequence for QPCR

| Oligos | Sequence (5’-3’) forward | Sequence (5’-3’) reverse |
|---|---|---|
| PARK2 | CACACCCCACCTCTGACAAG | CTAAGCAAATCACGTGGCGG |
| PINK1 | CGAAGCCATCTTGAACACAA | GTTGCTTGGGACCTCTCTTG |
| ANKRD1 | AGAACTGTGCTGGGAAGACG | GCCATGCCTTCAAAATGCCA |
| CTGF | CTCGCGGCTTACCGACTG | GGCTCTGCTTCTCTAGCCTG |
| CYR61 | AGCAGCCTGAAAAAGGGCAA | AGCCTGTAGAAGGGAAACGC |
| GAPDH | ACGGGAAGCTTGTCATCAAT | TGGACTCCACGACGTACTCA |

Supplementary table 2

## Small interfering RNA sequence for YAP and PINK1

| Oligos | Sequence (5’-3’) forward | Sequence (5’-3’) reverse |
|---|---|---|
| si YAP-1 | CACCUAUCACUCUCGAGAUdTdT | AUCUCGAGAGUGAUAGGUGdTdT |
| si YAP-2 | GCUCAUUCCUCUCCAGCUUdTdT | AAGCUGGAGAGGAAUGAGCdTdT |
| si YAP-3 | CUAAGCAUGAGCAGCUACAdTdT | UGUAGCUGCUCAUGCUUAGdTdT |
| Negative control | UUCUCCGAACGUGUCACGUTT | ACGUGACACGUUCGGAGAATT |
| si PINK1-1 | CCUAUGAAAUCUUCGGGCUdTdT | AGCCCGAACAUUUCAUAGGdTdT |
| si PINK1-2 | CGCAAAUGUGCUUCAUCUAdTdT | UAGAUGAAGCACAUUUGCGdTdT |
| Negative control | UUCUCCGAACGUGUCACGUTT | ACGUGACACGUUCGGAGAATT |

Supplementary table 3

The guide RNA sequence for PARK2 and YAP knockout

| Oligos | Sequence (5'-3') forward | Sequence (5'-3') reverse |
| --- | --- | --- |
| PARK2 sg RNA-1 | CACC GTCCTCCCCTATCAACCCCG | AAAC CGGGGTTGATAGGGGAGGAC |
| PARK2 sg RNA-2 | CACC G TACAACCGGTACCAGCAGTA | AAAC TACTGCTGGTACCGGTTGTA C |
| PARK2 sg RNA-3 | CACC GACAGGAACACACCGCTCCA | AAAC TGGAGCGGTGTGTTCCTGTC |
| YAP sg RNA-1 | CACCGTAATAGGCCAGTACTGATGC | AAACGCATCAGTACTGGCCTATTAC |
| YAP sg RNA-2 | CACCGACCCCCACTGGAGTAGTCTC | AAACGAGACTACTCCAGTGGGGGTC |
| YAP sg RNA-3 | CACCGTGTACCTCTGCCAGCAGGTT | AAACAACCTGCTGGCAGAGGTACAC |
